# Value of bowel preparation techniques for prostate MRI: a preliminary study

**DOI:** 10.1007/s00261-021-03046-3

**Published:** 2021-03-26

**Authors:** Cynthia Schmidt, Andreas M. Hötker, Urs J. Muehlematter, Irene A. Burger, Olivio F. Donati, Borna K. Barth

**Affiliations:** 1 Institute of Diagnostic and Interventional Radiology, University Hospital Zurich, Raemistrasse 100, 8091 Zurich, Switzerland; 2 Department of Nuclear Medicine, University Hospital Zurich, Raemistrasse 100, 8091 Zurich, Switzerland; 3 Department of Nuclear Medicine, Cantonal Hospital Baden, Im Ergel 1, 5404 Baden, Switzerland; 4 University of Zurich (UZH), Raemistrasse 71, 8006 Zurich, Switzerland

**Keywords:** Prostate MRI, Prostate cancer, Bowel preparation, Image quality

## Abstract

**Background:**

Bowel preparation before multiparametric MRI (mpMRI) of the prostate is performed widely, despite contradictory or no evidence for efficacy.

**Purpose:**

To investigate the value of hyoscine N-butylbromide (HBB), microenema (ME) and ‘dietary restrictions’ (DR) for artifact reduction and image quality (IQ) in mpMRI of the prostate.

**Study type:**

Retrospective.

**Population:**

Between 10/2018 and 02/2020 treatment-naïve men (median age, 64.9; range 39.8–87.3) who underwent mpMRI of the prostate were included. The total patient sample comprised of *n *= 180 patients, who received either HBB, ME, were instructed to adhere to DR, or received a combination of those measures prior to the MR scan.

**Field strength/sequence:**

T2-weighted imaging (T2w), diffusion-weighted imaging (DWI), and dynamic contrast-enhanced MRI (DCE-MRI) scanned on two 3T systems.

**Assessment:**

A radiologist specialized in urogenital imaging (R1) and a senior radiology resident (R2) visually assessed IQ parameters on transversal T2w, DWI and ADC maps on a 5-point Likert-like scale.

**Statistical tests:**

Group comparison between IQ parameters was performed on reader level using Kruskal–Wallis and Mann–Whitney *U* tests. Binary univariate logistic regression analysis was used to assess independent predictors of IQ. Interrater agreement was assessed using Intraclass Correlation Coefficient (ICC).

**Results:**

‘DWI geometric distortion’ was significantly more pronounced in the HBB+/ME−/DR− (R1, 3.6 and R2, 4.0) as compared to the HBB−/ME+/DR− (R1, 4.2 and R2, 4.6) and HBB+/ME+/DR− (R1, 4.3 and R2, 4.7) cohort, respectively. Parameters ‘DWI IQ’ and ‘Whole MRI IQ’ were rated similarly by both readers. ME was a significant independent predictor of ‘good IQ’ for the whole MRI for R1 [*b* = 1.09, OR 2.98 (95% CI 1.29, 6.87)] and R2 [*b* = 1.01, OR 2.73 (95% CI 1.24, 6.04)], respectively.

**Data conclusion:**

ME seems to significantly improve image quality of DWI and the whole mpMRI image set of the prostate. HBB and DR did not have any benefit.

## Introduction

Multiparametric MRI (mpMRI) has become an invaluable tool in assessment of men at risk for prostate cancer [1, 2]. Superior MRI image quality (IQ) is indispensable for the radiologist to deliver an accurate diagnosis [3]. The importance of scan quality for prostate cancer detection is reflected in the recent development of a new scoring system for the evaluation of multiparametric prostate MRI IQ, the Prostate Imaging Quality (PI-QUAL) score [4], which rates diagnostic quality by means of technical and visual assessment criteria. Scan quality can be impaired by artifacts, such as motion or susceptibility. Several patient preparation techniques have been proposed to mitigate those artifacts [5–7].

Motion artifacts can lead to ghosting, blurring and a reduced signal-to-noise ratio, particularly in images with a long scan time, like T2-weighted images [8, 9]. A superior IQ is particularly valuable for discerning fine anatomical structures, for example the border of nodules within the transitional zone when differentiating between a PI-RADS 2 and 3 score [10]. In this context, antispasmodic agents such as hyoscine N-butylbromide (HBB) are widely used for suppression of bowel peristalsis. However, study results regarding the use of HBB are equivocal, as some authors did find positive effects on artifact reduction and/or IQ [7, 11], while others did not [9, 12].

Gas or stool may distend the rectum, introduce susceptibility artifacts and deform the dorsal prostate contour through geometric distortion [13, 14]. This issue may be particularly unfavorable, as the majority of prostate carcinomas arise within the peripheral zone [15]. Diffusion-weighted imaging (DWI) suffers most from these types of artifacts, notably at higher field strengths [16]. Hence, measures to evacuate the rectum before the MRI exam seem reasonable. Studies investigating the use of a preparatory microenema (ME) before scanning revealed that stool/gas and related artifacts in DWI can be reduced significantly [17, 18]. Lim et al. [19] even showed that the amount of stool correlates positively with motion artifacts on T2w. However, the effect either does not [19] or only modestly translate into better IQ itself [18], indicating the complexity of the topic, as solely emptying the rectum or reducing artifacts does not seem to be sufficient. Imposing fasting measures prior to the MR scan is another hypothesis on how to reduce bowel peristalsis. However, the topic has not been investigated extensively up to date. The discrepancy of the results in the literature on HBB and ME most likely explains why there was no consensus reached regarding this topic in current guidelines such as the “Prostate Imaging and Reporting Data System (PI-RADS) v2.1” (10). Finally, despite the fact that HBB and ME are very often used simultaneously, no study investigated the effect of those techniques in combination or the specific contribution of one intervention alone regarding IQ in prostate MRI.

Therefore, the purpose of this study was to investigate the value of HBB, ME, DR in combination and alone for artifact reduction and IQ in prostate MRI.

## Materials and methods

### Study population

The regional ethics committee approved this retrospective study and written general informed consent on usage of data for research purposes was obtained from all patients.

At our institution patients referred for a clinically indicated mpMRI of the prostate are randomly assigned to one of the two identical 3T (Tesla) MRI scanners (MR Scanner 1 and MR Scanner 2), depending on slot availability. Due to organizational restrictions, ME prior to the MRI exam is given to patients scanned on MR Scanner 1 only. To test bowel preparation techniques in the clinical setting, we introduced HBB on 03-27-2019 (Time Point 1[Tp1]) and DR on 10–01-2019 (Time Point 2[Tp2]) for all patients without contraindications.

**Fig. 1:**
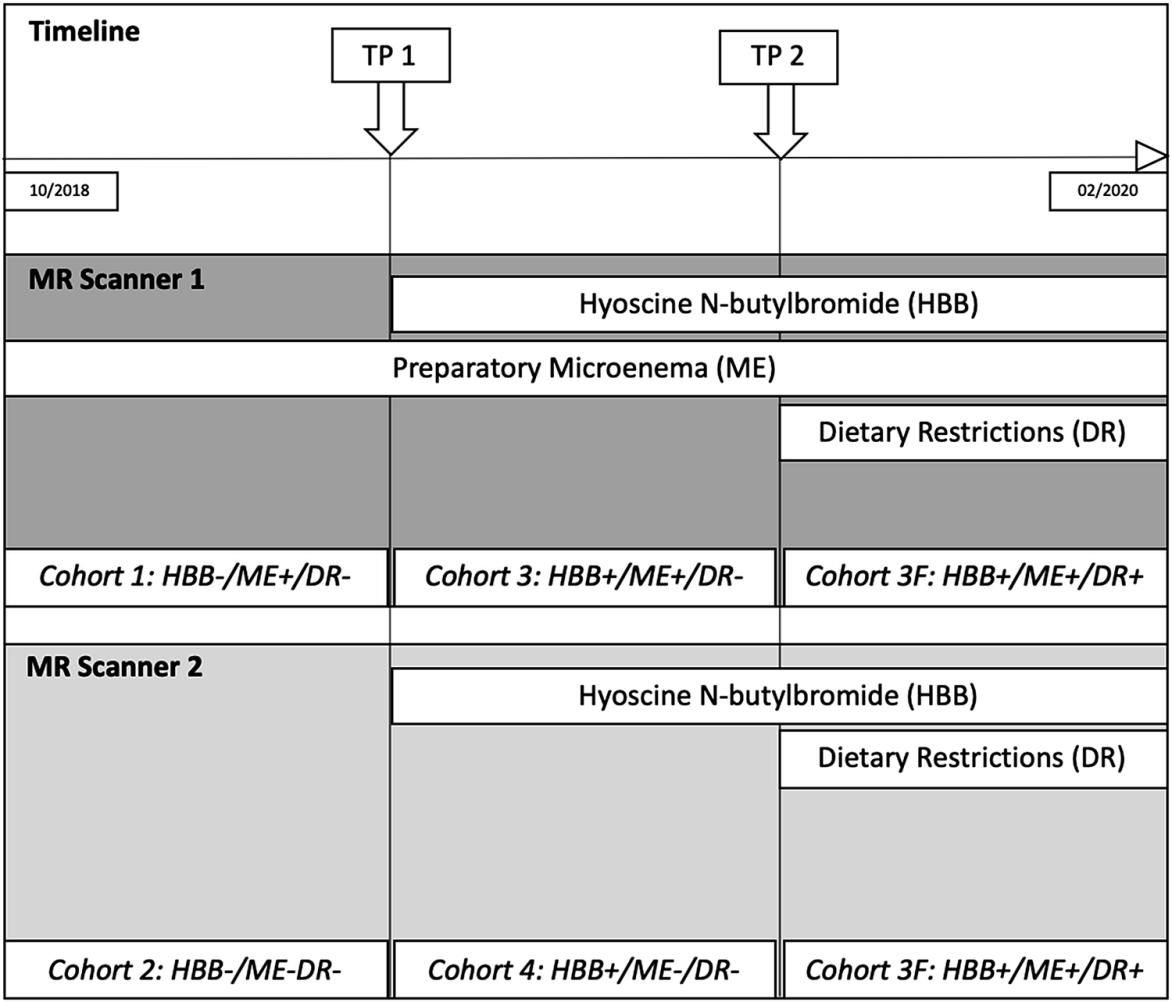
Timeline for patient inclusion on MR Scanner 1 and 2. Time Point (TP)

**Fig. 2:**
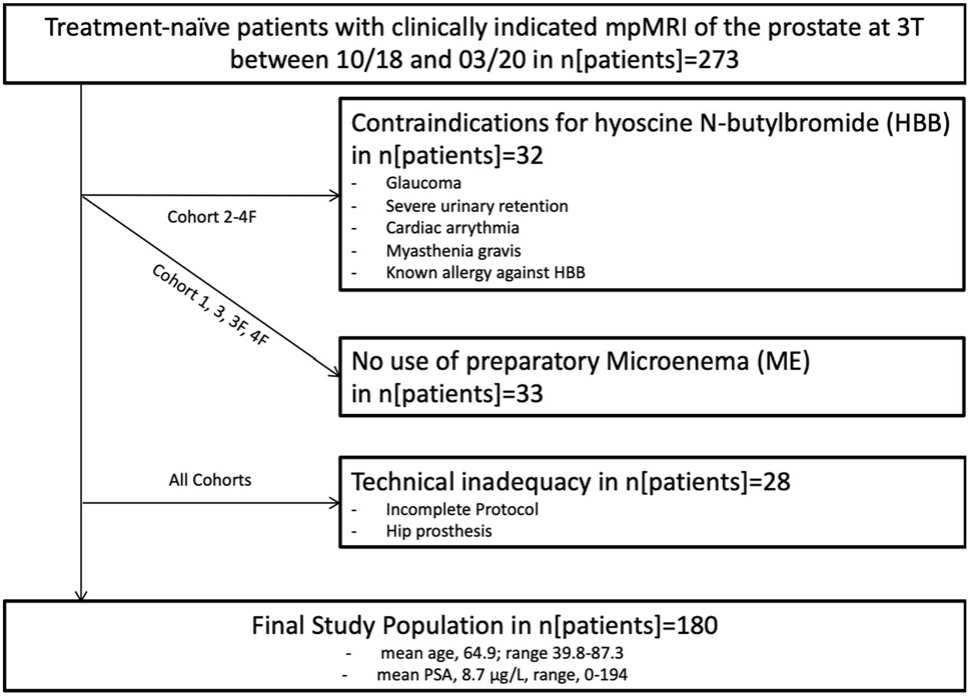
Flow chart diagram showing the inclusion process

For this study, all treatment-naïve men over the age of 18 years undergoing mpMRI of the prostate at 3T were deemed eligible (*n* = 273). The timeline for patient inclusion on MR Scanner 1 and 2 is shown in Fig. 1. We excluded patients who did not receive ME on Sc1 before the MR exam and/or those who were scanned after Tp1 and who had contraindications for HBB (*n* = 65) using the institutional radiological information system (RIS), patients with an incomplete scan protocol (*n* = 6) and patients with hip implants (*n* = 22). All patients who were scanned after Tp2 adhered to the imposed DR before the MR scan, which was assessed by a questionnaire prior to the MRI exam. The inclusion process was continued, until a quota of 30 patients per cohort was met. The final study population comprised of 180 patients [mean age 64.9 years (range 39.8–87.3 years); mean PSA: 8.7 μg/L (range 0–194 μg/L), mean prostate volume: 55.1 mL (range 15.6–65 mL) and mean PSA density: 0.18 (range 0–3.7)]. The inclusion process is shown in Fig. 2. The cohorts were named according to the used bowel preparation technique:

Cohort 1 (no HBB, ME, no DR): HBB−/ME+/DR−

Cohort 2 (no HBB, no ME, no DR): HBB−/ME−/DR−

Cohort 3 (HBB, ME, no DR): HBB+/ME+/DR−

Cohort 4 (HBB, no ME, no DR): HBB+/ME−/DR−

Cohort 3F (HBB, ME, DR): HBB+/ME+/DR+

Cohort 4F (HBB, no ME, DR): HBB+/ME−/DR+

### MRI technique

Images were acquired on two 3T whole-body MRI systems (MAGNETOM Skyra, SIEMENS Healthcare®, Erlangen, Germany). A 2 × 30-channel phased-array coil was used for signal reception at MR Scanner 1, while an 18-channel phased-array coil was used at MR Scanner 2.

T2-weighted turbo-spin echo images (T2w) were obtained in three planes (transverse, coronal and sagittal), covering the whole prostate including the seminal vesicles; however, only the transversal images were evaluated in this study setting. Diffusion-weighted images (DWI) were acquired with identical orientation as the transverse T2w images. Apparent diffusion coefficient (ADC) parametric maps were calculated using a monoexponential fit based on three obtained b-values (100, 600, 1000 s/mm^2^). A high b-value (1400 s/mm^2^) was calculated based on a standard monoexponential fit. Dynamic contrast-enhanced images (DCE-MRI) were also obtained with identical orientation as the transverse T2w images using a 3D T1w spoiled gradient-echo pulse sequence with a temporal resolution of < 8 s. Gadoterate meglumine (Dotarem; Guerbet, Darmstadt, Germany) was used as contrast agent with a dose of 0.1 mmol/kg bodyweight. Injection was performed with an automated MR injection system (Spectris Solaris EP, MEDRAD MR Injector, Bayer HealthCare LCC, Whippany NJ) at a flow rate of 2 mL/sec. Basic scan parameters are shown in Table 1.

**Table 1:** Basic scan parameters

	T2w	DWI	DCE-MRI
*b*-value (s/mm^2^)	–	100, 600, 1000 1400 (Calc)	–
Number of averages	2	2, 4, 8	–
Imaging planes per sequence	3	1	1
Typical TR (ms)	8100	12,100	5.08
Typical TE (ms)	92	86	1.8
Echo train length	24	45	1
Field of view (mm)	160 × 160	160 × 83	230 × 230
Reconstruction matrix (mm)	640 × 640	200 × 104	224 × 224
In-plane resolution	0.25 × 0.25	0.80 × 0.80	1.03 × 1.03
Slice thickness (mm)	3	3	3
Gap between slices (mm)	0	0.3	0
Acquisition time (min)	03:16	05:14	02:46

### Bowel preparation techniques

1. *Microenema (ME):* Patients scanned on MR Scanner 1 received a liquid, preparatory microenema (Freka Clyss®, Fresenius Kabi, Germany) in a 133 mL bottle and were briefed on how to use the product. They were instructed to apply it immediately before the MRI exam and to evacuate the rectum if necessary. Although actively recommended, the application of ME was facultative and it was handed out to those patients only, who were willing to use it. The patients had to report if application was unsuccessful.
2. *Hyoscine N-butylbromide/Butylscopolamine (HBB):* Patients scanned after Tp1 were administered 20 mg of an antispasmodic agent (Buscopan®, Boehringer Ingelheim, Germany) as a single-dose intravenous (i. v.) injection by the MR technician immediately before the acquisition of the transversal T2w. MR technicians were instructed to keep the time between the injection and the start of the acquisition as short as possible. Contraindications were glaucoma, severe urinary retention, cardiac arrythmia, myasthenia gravis and known allergy against HBB.
3. *Dietary Restrictions (DR):* All patients scheduled for an MR scan after Tp2 were instructed to fast 6 h prior to the exam. Solely consumption of water was allowed. The patients had to report if they did not adhere to the DR.

### Readout

The MRI exams of the 180 patients were stored within the institutional Picture Archiving and Communicating System (IMPAX®, Agfa Healthcare, Germany). A radiologist specialized in urogenital imaging (initials blinded for review) with 10 years of experience in prostate MRI reporting (R1) and a radiology resident with special interest in prostate imaging (initials blinded for review) with 4 years of experience in prostate MRI reporting (R2) performed the qualitative readout. Parameters regarding IQ, artifacts and the presence of stool/gas within the rectum were assessed in one readout session. Readers were blinded to the type of bowel preparation technique. Qualitative parameters and rating scales are shown in Table 2.

**Table 2:** Qualitative parameters and rating scales

	Description	Scale
T2w		
Anatomic detail	Visualization of the capsule and organ borders, the ejaculatory ducts and seminal vesicles, delineation of the zonal anatomy, distinction of nodules in the transitional zone and depiction of the neurovascular bundle	1–5*
Ghosting artifacts	Abnormal extension/multiplication of the anatomic structure along the phase-encoding direction	1–5**
Image quality (IQ)	Overall impression of image quality (IQ), encompassing all aspects of the sequence, including artifacts	1–5^†^
DWI		
Geometric distortion	Morphologic distortion of the gland anatomy in relation to T2w	1–5**
Ghosting artifacts	Abnormal extension/multiplication of the anatomic structure along the phase-encoding direction	1–5**
Image quality (IQ)	Overall impression of image quality (IQ), encompassing all aspects of the sequence, including artifacts	1–5^†^
ADC		
Images diagnostic	Is the quality of the ADC sufficient for diagnostic assessment	Yes/No
Whole MRI		
Image quality (IQ)	Overall impression of image quality (IQ), encompassing all aspects of the whole MRI exam, including T2w, DWI, ADC and DCE-MRI	1–5^†^
Image set diagnostic	Is the quality of the whole MRI exam sufficient for diagnostic assessment	Yes/No
Stool/gas		
Presence	Amount of Stool/Gas within the rectum	1–3^††^

*1… non-diagnostic, structures cannot be evaluated; 2… poor visualization; heavily blurred appearance of structures; 3… moderate visualization, moderate blurring; 4… good delineation, slight blurring; 5… excellent visualization, sharp delineation

**1… severe artifacts; 2…considerable artifacts; 3…moderate artifacts; 4…mild artifacts; 5…no artifacts

^†^1…poor; 2…below average; 3…average; 4…above average; 5…excellent

^††^1…no stool/gas; 2…minimal amount; 3…large amount

### Statistical analysis

Demographic variables were summarized as means with ranges and absolute numbers with percentages. Categorical variables were summarized as absolute figures. Shapiro–Wilk test was used to assess the distribution of data.

Kruskal–Wallis test was used to assess the central tendency of the IQ parameters between cohorts 1-4F. Frequency distributions of dichotomous variables were reported as counts and proportions.

Pearson’s Chi-square test (*χ*^2^) was used to test the relationship between the predictor- and outcome variable/s. Binary univariate logistic regression analysis was used to assess the influence of the predictor variables HBB and ME on the artificially created, dichotomous outcome variable ‘image quality’. The outcome variable was defined as follows: Likert-like scores 1–3 derived from the IQ parameter ‘whole MRI IQ’ were defined as ‘low-moderate’ image quality and scores 4 and 5 as ‘good’ image quality and dummy-coded accordingly. Interrater agreement for the IQ parameters was assessed using intraclass correlation coefficient (ICC, two-way random), including 95% confidence intervals (CI). The ICC values were interpreted as follows: 0–0.40, poor agreement; 0.41–0.58, fair agreement; 0.59–0.75, good agreement; 0.76–1.0, excellent agreement [20].

The statistical analysis was performed for both readers, independently. A *p* value of < 0.05 was considered statistically significant. Statistical analysis was performed with IBM SPSS statistical software (SPSS version 21; Chicago, Il).

## Results

### Cohort comparison of ordinally scaled IQ parameters

For R1, DWI geometric distortion was significantly less pronounced in the HBB+/ME+/DR− as compared to the HBB−/ME−/DR− (4.3 and 3.5, *p* < 0.05) and HBB+/ME−/DR− (4.3 and 3.6, *p* < 0.05) cohorts. For R2, DWI geometric distortion was significantly more pronounced in the HBB+/ME−/DR− as compared to the HBB−/ME+/DR− (4 and 4.6, *p* < 0.05) and HBB+/ME+/DR− (4 and 4.7, *p* < 0.05) cohorts. DWI IQ was rated similarly by both readers.

**Fig. 3:**
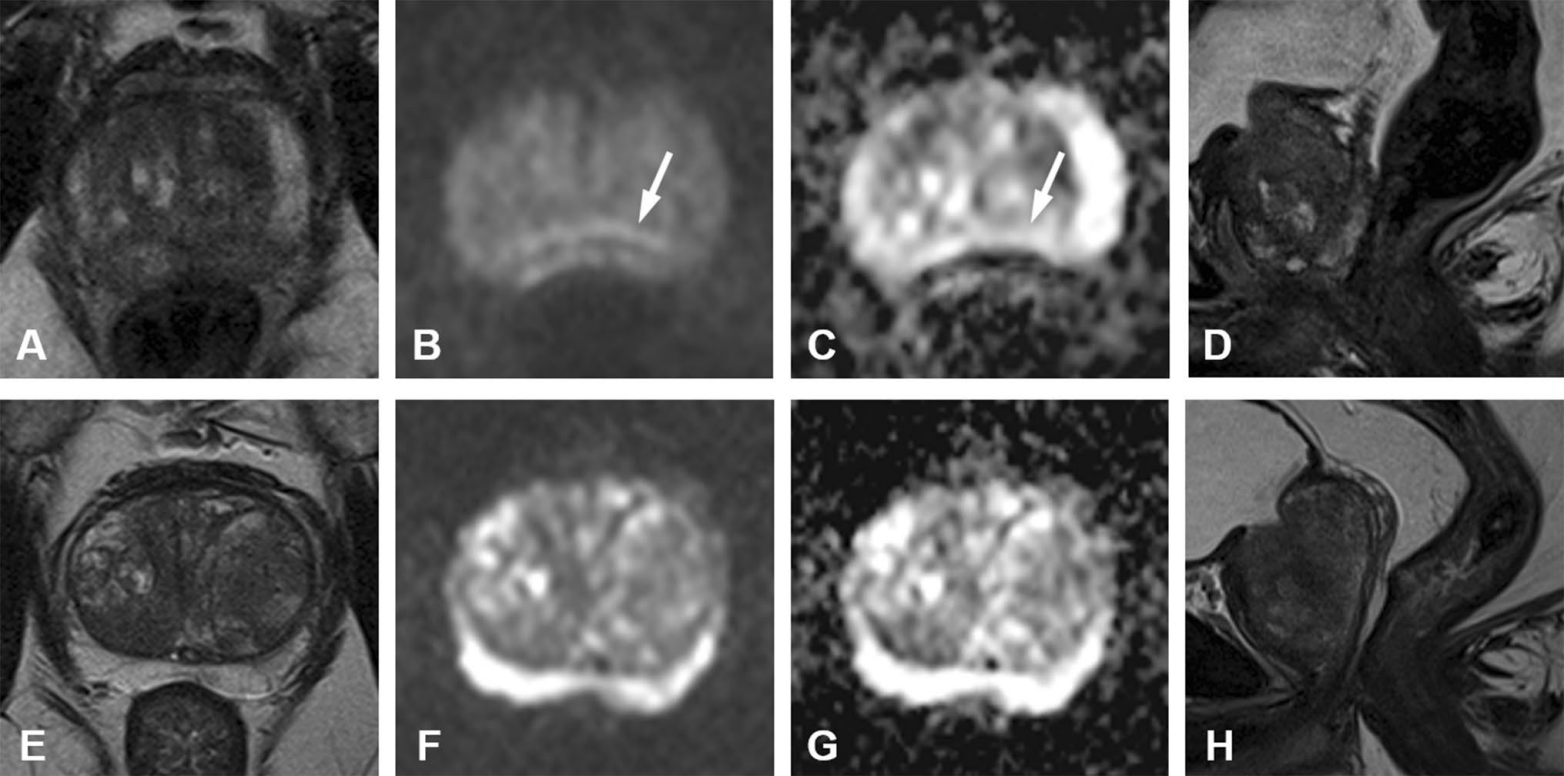
Multiparametric MRI of the prostate of a 70-year old patient (P1) within the cohort HBB−/ME−/DR− (**a**–**d**) compared to a 55-year old patient (P2) within the cohort HBB−/ME+/DR− (**e**–**h**). P2 applied microenema (ME) before MRI scan, P1 did not. Both patients did not receive hyoscine *N*-butylbromide (HBB). A transverse and sagittal T2w, a DWI b-1000 and the corresponding ADC map are shown. Note the presence of susceptibility artifacts (white arrows) on the posterior border of the prostate on the DWI b-1000 and ADC map in P1 without ME (**b**, **c**), which presumably explained due to an increased amount stool/gas in the rectum, particularly well visualized on the sagittal T2w (**d**), as compared to P2 (**h**). Also note the relatively increased blurring on the T2w of P1 (**a**), as compared to the P2 (**e**)

For R1 and R2, presence of stool/gas was significantly higher (*p* < 0.05) in the HBB+/ME−/DR− (2.4 and 2.7) as compared to the HBB−/ME+/DR− (1.5 and 1.8) and HBB+/ME+/DR− (1.4 and 1.9) cohorts (Fig. 3). Moreover, for R1 presence of stool/gas was significantly higher (*p* < 0.05) in the HBB+/ME−/DR+ as compared to the HBB−/ME+/DR− (2.1 and 1.5) and HBB+/ME+/DR− (2.1 and 1.4) cohorts. R2 rated the presence of stool/gas similarly.

For R1 whole MRI IQ was significantly higher (*p* < 0.05) in the HBB+/ME+/DR− (4.2) as compared to the HBB+/ME−/DR− (3.5) cohorts. For R2 differences in whole MRI IQ did not reach statistical significance.

There was no statistically significant difference (*p* > 0.05) of qualitative IQ parameters neither between cohort 3 (HBB+/ME+/DR−) and 3F (HBB+/ME+/DR+), nor between cohort 4 (HBB+/ME−/DR−) and 4F (HBB+/ME−/DR+), for R1 and R2 respectively. Only for R2 DWI geometric distortion was significantly more pronounced in the HBB+/ME−/DR+ as compared to the HBB−/ME+/DR− (4 and 4.6, *p* < 0.05) and HBB+/ME+/DR− (4 and 4.7, *p* < 0.05) cohorts.

No statistically significant difference (p > 0.05) was found for the remaining qualitative parameters. A detailed overview of the cohort 1-4F comparisons and ranking of selected IQ parameters is shown in Table 3.

**Table 3:** Cohort 1-4F comparisons and ranking of selected Image Quality (IQ) parameters

Cohort	Reader 1 (R1)							Reader 2 (R2)							Ranking R1	Ranking R2
	HBB− ME+	HBB− ME−	HBB+ME+	HBB+ME−	HBB+ME+DR+	HBB+ME− DR+	Mean score*	HBB− ME+	HBB− ME−	HBB+ME+	HBB+ME−	HBB+ ME+DR+	HBB+ME− DR+	Mean score*		
IQ parameter	DWI geometric distortion															
HBB−/ME+/DR−	x	> 0.05	> 0.05	> 0.05	> 0.05	> 0.05	4.2	x	> 0.05	> 0.05	**0.022**	> 0.05	**0.017**	4.6	2	2
HBB−/ME−/DR−	x	x	**0.011**	> 0.05	> 0.05	> 0.05	3.5	x	x	> 0.05	> 0.05	> 0.05	> 0.05	4.1	6	4
HBB+/ME+/DR−	x	x	x	**0.016**	> 0.05	> 0.05	4.3	x	x	x	**0.004**	> 0.05	**0.003**	4.7	1	1
HBB+/ME−/DR−	x	x	x	x	> 0.05	> 0.05	3.6	x	x	x	x	> 0.05	> 0.05	4.0	5	*5*
HBB+/ME+/DR+	x	x	x	x	x	> 0.05	3.8	x	x	x	x	x	> 0.05	4.3	3	3
HBB+/ME−/DR+	x	x	x	x	x	x	3.8	x	x	x	x	x	x	4.0	5	*5*
IQ parameter	DWI image quality															
HBB−/ME+/DR−	x	> 0.05	> 0.05	> 0.05	> 0.05	> 0.05	4.0	x	> 0.05	> 0.05	**0.009**	> 0.05	> 0.05	3.8	2	1
HBB−/ME−/DR−	x	x	> 0.05	> 0.05	> 0.05	> 0.05	3.5	x	x	> 0.05	> 0.05	> 0.05	> 0.05	3.2	*4*	4
HBB+/ME+/DR−	x	x	x	**0.01**	> 0.05	> 0.05	4.1	x	x	x	> 0.05	> 0.05	> 0.05	3.7	1	2
HBB+/ME−/DR−	x	x	x	x	> 0.05	> 0.05	3.5	x	x	x	x	> 0.05	> 0.05	3.0	*4*	6
HBB+/ME+/DR+	x	x	x	x	x	> 0.05	3.8	x	x	x	x	x	> 0.05	3.5	*3*	3
HBB+/ME−/DR+	x	x	x	x	x	x	3.8	x	x	x	x	x	x	3.1	*3*	5
IQ parameter	Presence Stool/Gas															
HBB−/ME+/DR−	x	**0.001**	> 0.05	** < 0.001**	**0.02**	**0.18**	1.5	x	**0.032**	> 0.05	** < 0.001**	> 0.05	**0.001**	1.8	2	1
HBB−/ME−/DR−	x	x	** < 0.001**	> 0.05	> 0.05	> 0.05	2.3	x	x	> 0.05	> 0.05	> 0.05	> 0.05	2.4	5	3
HBB+/ME+/DR−	x	x	x	** < 0.001**	> 0.05	**0.012**	1.4	x	x	x	** < 0.001**	> 0.05	**0.007**	1.9	1	*2*
HBB+/ME−/DR−	x	x	x	x	**0.003**	> 0.05	2.4	x	x	x	x	** < 0.001**	> 0.05	2.7	6	5
HBB+/ME+/DR+	x	x	x	x	x	> 0.05	1.6	x	x	x	x	x	**0.007**	1.9	3	*2*
HBB+/ME−/DR+	x	x	x	x	x	x	2.1	x	x	x	x	x	x	2.6	4	4
IQ parameter	Whole MRI image quality															
HBB−/ME+/DR−	x	> 0.05	> 0.05	> 0.05	> 0.05	> 0.05	4.0	x	> 0.05	> 0.05	> 0.05	> 0.05	> 0.05	3.5	2	1
HBB−/ME−/DR−	x	x	> 0.05	> 0.05	> 0.05	> 0.05	3.6	x	x	> 0.05	> 0.05	> 0.05	> 0.05	3.1	4	4
HBB+/ME+/DR−	x	x	x	**0.007**	> 0.05	> 0.05	4.2	x	x	x	> 0.05	> 0.05	> 0.05	3.3	1	*2*
HBB+/ME−/DR−	x	x	x	x	> 0.05	> 0.05	3.5	x	x	x	x	> 0.05	> 0.05	3.0	5	5
HBB+/ME+/DR+	x	x	x	x	x	> 0.05	3.8	x	x	x	x	x	> 0.05	3.3	*3*	*2*
HBB+/ME−/DR+	x	x	x	x	x	x	3.8	x	x	x	x	x	x	3.2	*3*	3

*HBB* Hyoscine *N*-butylbromide, *ME* Preparatory Microenema, *DR* dietary restrictions

Statistically significant results between groups (*p* < 0.05) are highlighted in bold

*Mean Score for DWI Geometric Distortion, DWI Image Quality and Whole MRI Image Quality: 1 best, …, 5 worst

Mean Score for Presence Stool/Gas: 1…high amount of stool, …, 3 no stool

^†^Rank for DWI Geometric Distortion, DWI Image Quality and Whole MRI Image Quality: 1 best, …, 6 worst

Rank for Presence Stool/Gas: 1…worst, …, 6 best

**Fig. 4:**
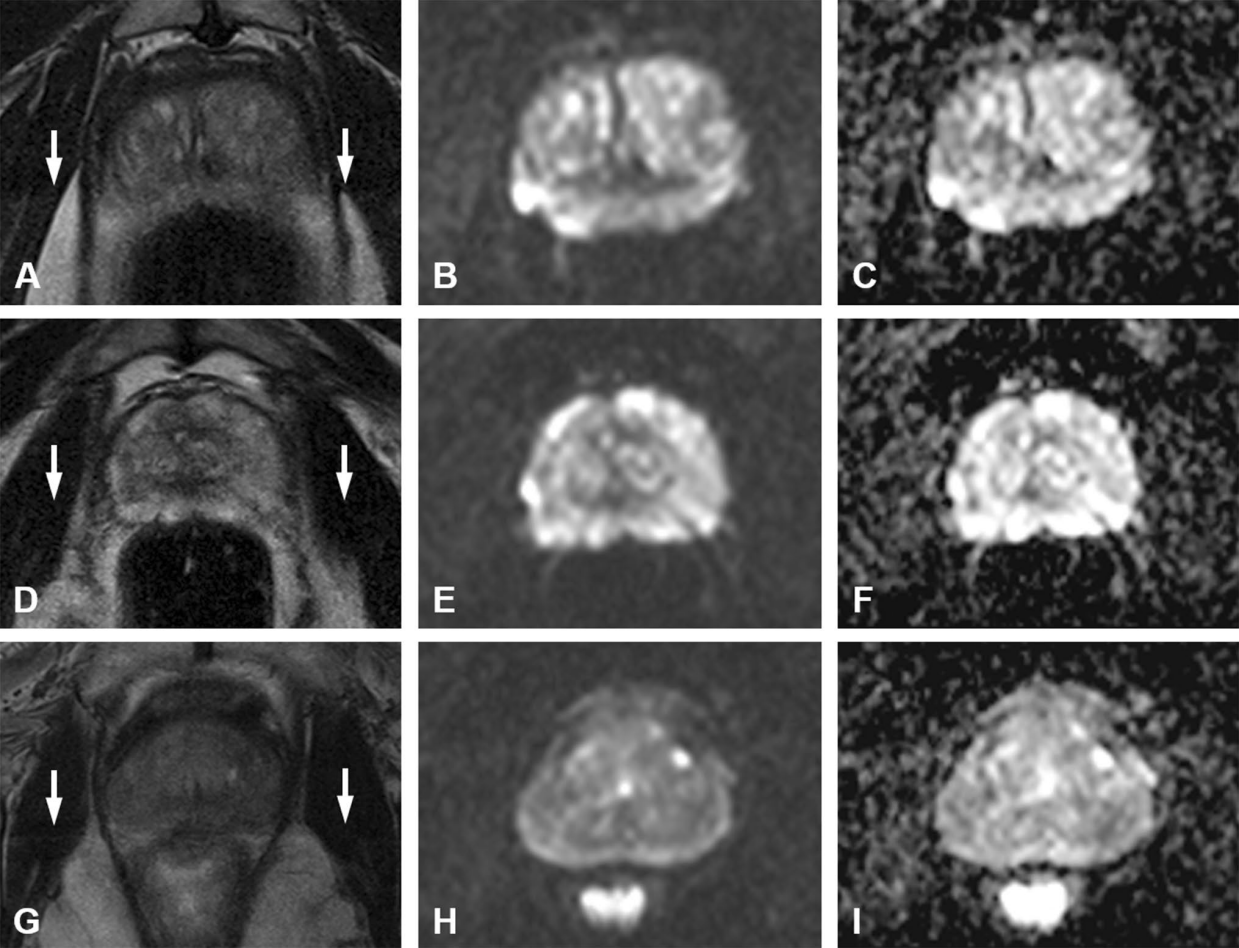
Multiparametric MRI of the prostate of a 63-year old patient (P1) within the cohort HBB−/ME−/DR− (**a**–**c**), a 63-year old patient P2 within the cohort HBB+/ME−/DR− (**d**–**f**) and a 64-year old patient P3 within the cohort HBB+/ME−/DR+ (**g**–i). In this setting no patient received microenema (ME). Note the presence of ghosting artifacts (white arrows) on T2w images across all setups- i. e. with and without hyoscine *N*-butylbromide (HBB) and/or dietary restrictions (DR). Moreover, all T2w images are blurred in a similar way

Figure 4 shows ghosting artifacts and image blurring which were found across all cohorts with and without HBB and/or DR alike.

### Proportions comparison of binary scaled IQ parameters

For R1, in *n* = 4/30 (13.3%) of HBB−/ME−/DR− patients, (i) the ADC map and (ii) the whole MRI exam were rated not diagnostic. For R2, in *n* = 2/30 (6.7%) of HBB+/ME−/DR− patients the ADC map was rated not diagnostic and in *n* = 1/30 (3.3%) of HBB+/ME−/DR− patients the whole MRI exam was rated not diagnostic. Cumulatively, in n = 5/30 (16.7%) of HBB−/ME−/DR− patients the whole MRI exam was rated not diagnostic. Cumulatively, whole MRI exam was rated not diagnostic when ME was not used in *n* = 10/30 (33.3%) and when HBB was not used in *n* = 6/30 (20.0%). A detailed overview of the proportions comparison is shown in Table 4.

**Table 4:** Overview of the proportions comparison

Cohort	Reader 1 (R1)		Reader 2 (R2)		Cumulative (R1, R2)							
	*n*	*n* (%*)	*n*	*n* (%*)	*n*	*n* (%*)	ME used	No ME	HBB used	No HBB	DR	No DR
IQ parameter	ADC images NOT diagnostic											
HBB−/ME+/DR−	0	0	0	0	0	0	* n*					
HBB−/ME−/DR−	4	13.3	0	0	4	13.3	5	9	10	4	6	8
HBB+/ME+/DR−	1	3.3	0	0	1	3.3	* n* (%*)					
HBB+/ME−/DR−	1	3.3	2	6.7	3	10	16.7	30	33.3	13.3	20	26.7
HBB+/ME+/DR+	3	10	1	3.3	4	13.3						
HBB+/ME−/DR+	2	6.7	0	0	2	6.7						
IQ parameter	Whole MRI images NOT diagnostic											
HBB−/ME+/DR−	1	3.3	0	0	1	3.3	* n*					
HBB−/ME−/DR−	4	13.3	1	3.3	5	16.7	6	10	10	6	7	9
HBB+/ME+/DR−	1	3.3	0	0	1	3.3	* n* (%*)					
HBB+/ME−/DR−	1	3.3	1	3.3	2	6.7	20	33.3	33.3	20	23.3	30
HBB+/ME+/DR+	3	10	1	3.3	4	13.3						
HBB+/ME−/DR+	3	10	0	0	3	10						

*HBB* Hyoscine *N*-butylbromide, *ME* preparatory microenema, *DR* dietary restrictions

*Percentage per Cohort (*n* = 30)

### Predictors of image quality

ME was a statistically significant predictor of ‘good’ IQ (score 4, 5) for whole MRI IQ for R1 (*b* = 1.09, OR 2.98 [95% CI 1.29, 6.87]) and R2 (*b* = 1.01, OR 2.73 [95% CI 1.24, 6.04]), respectively. Stepwise inclusion of (i) HBB and (ii) a combination of HBB and ME did not significantly improve the model, neither for R1 (p[Block i] = 0.535 and p[Block ii] = 0.359) nor for R2 (p[Block i] = 0.165 and p[Block ii] = 0.706). A detailed overview of the logistic regression analysis is shown in Table 5.

**Table 5:** Coefficients of the Model predicting ‘good’ Image Quality^†^

	*b* [SE]	95% CI for odds ratio			
		Lower	OR	Upper	
Reader 1					
HBB	− 0.26 [0.42]	0.34	0.77	1.74	
ME	1.09 [0.43]*	1.29	2.98	6.87	
Constant	0.54 [0.34]	n. a	1.71	n. a	
	*R*^2^ = *.08 (Hosmer&Lemeshow), 0.06 (Cox&Snell), 0.09 (Nagelkerke), Model X*^*2*^ = *7.33, p* < 0.05; *p < 0*.05*				
Reader 2					
HBB	− 0.55 [0.40]	0.26	0.58	1.26	
ME	1.01 [0.40]*	1.24	2.73	6.04	
Constant	− 0.93 [0.36]	n. a	0.34	n. a	
	*R*^2^ = *.14 (Hosmer&Lemeshow), 0.07 (Cox&Snell), 0.09 (Nagelkerke), Model X*^*2*^ = *8.27, p* < 0.05; *p < 0*.05*				

Hyoscine *N*-butylbromide (HBB); Preparatory Microenema (ME)

^†^Defined as scores 4 and 5 for the parameter whole MRI Image Quality (IQ)

### Interrater agreement (ICC)

Mean ICC for all IQ parameters was 0.46 [range 0.27–0.79].

## Discussion

In our study, ME had a positive impact on reducing artifacts and improving IQ. Antispasmodic pre-medication with HBB and DR or combinations thereof appear to show no positive or synergic effect.

Bowel peristalsis may induce movement of pelvic organs, eventually leading to artifacts depending on the direction of k-space sampling [8, 9]. Our study results showed no benefit with HBB, neither when used exclusively, nor in combination with ME and or DR. We observed a positive trend toward better delineation of anatomic detail and less motion artifacts on T2w sequences for R1 only; however, the difference was not statistically significant. These results are in line with Wagner [9] and Roethke et al. [12]. Still, the data must be interpreted with caution due to comparably lower field strength (1.5T) used by Wagner et al. [9] in their study setting. Roethke et al. [12] assessed IQ parameters on the level of the whole MRI exam, and not based on a single sequence. There are two studies [7, 11] which are methodologically and technically similar to our study setup, demonstrating higher IQ and lower motion-related artifacts with HBB in T2w images. This might in part be explained by a different dose compared to our study setup. While we injected 20 mg of HBB, Slough [11] and Ullrich et al. [7] used a dose of 40 mg. However, despite intense pharmacodynamic and -kinetic investigation of the effects of HBB on bowel peristalsis [21], it is still not clearly established whether to use 20 or 40 mg in prostate imaging. Some MRI and CT studies suggest that the injection of 20 mg i. v. may be sufficient [8, 22–24]. Also results from a CT colonography study confirm that 20 mg of HBB i. v. significantly improved colonic distension without further improvement when increasing the dose to 40 mg [25]. Furthermore, empirically raising the dose to 40 mg HBB remains problematic due to associated side-effects, which may be significantly underestimated, as reported by Johnson et al. [8]. To summarize, whether the dose of 20 mg gives a sufficient explanation for the discrepancy of the available data remains unclear. We excluded patients with contraindications such as a history of glaucoma, severe urinary retention and cardiac arrythmia. However, studies have reported that it might be feasible to acknowledge only unstable cardiac disease as a contraindication and instead brief patients in advance about potential ocular symptoms, so they can seek medical help in the case of symptoms. This is due to the fact that particularly patients with new/undiagnosed cases of glaucoma have been described to be those truly at risk of acute angle glaucoma [26]. Hence, HBB side-effects might only be relevant to a small minority of patients. Based on (i) a superior potency for suppression of bowel peristalsis and (ii) a more favorable side-effect profile due to a lack of anticholinergic properties, glucagon may be a viable alternative [27]. Gutzeit et al. reported the best efficacy and longest duration of bowel peristalsis suppression for a combination of intramuscularly injected HBB and i. v. injected Glucagon, allegedly due to synergistic effects [28].

The bowel is subject to spontaneous phasic activity [29] which may be amplified by ingestion of food/coffee through initiation of propulsive peristaltic waves. Theoretically, fasting prior to the MRI exam should reduce this activity. However, in our study setting imposed DR did not add incremental value to (i) HBB alone or (ii) HBB and ME combined. Moreover, Reader 2 rated IQ in the HBB+/ME−/DR+ cohort significantly worse, as compared to the cohort using ME in combination with DR or with HBB and DR combined. Nevertheless, as bowel peristalsis may be influenced through different interacting mechanisms, the authors believe that—despite not reaching statistical significance—inhibition of bowel peristalsis may still be useful in prostate MRI, as also indicated by Roethke et al. [12].

Rectal distension through stool or gas has been shown to significantly increase geometric distortion of the prostate gland [14]. This effect is particularly pronounced in echoplanar imaging (EPI) and on higher field strengths [8, 9]. Two studies [17, 18] demonstrated that ME may reduce gas-related artifacts significantly in DWI. Our data confirms these results. However, while Coskun et al. [18] found only moderately positive effects in terms of artifact reduction in one of two readers, our results show stronger evidence in support of ME. The increase in DWI IQ translated into significantly better perception of overall IQ of the whole mpMRI exam and logistic regression analysis revealed ME as the single significant predictor of ‘good’ whole MRI IQ. Using ME before prostate MRI increased the chance for having ‘good’ whole MRI IQ by almost 3 times independently in both readers (OR R1, 2.98 and R2, 2.73). In line with our results, Lim et al. [19] found less stool in patients who used ME and the amount of stool did correlate with motion artifacts on T2w. However, DWI IQ did not improve with the use of ME. This is perhaps rooted in the low study power [19], as only a minority of patients in their study (*n* = 32) in the non-enema group had moderate or large amounts of stool, as opposed to our corresponding patient cohort (mean stool score 2.44/3). In addition, we observed a positive effect of ME on rectal evacuation independently of the concurrently used HBB and/or DR. According to the available literature, ME seemingly has a positive effect on rectum evacuation and it has the potential to significantly decrease susceptibility artifacts on DWI [5, 17]. Yet, it does not exert enough effect on bowel peristalsis in order to improve IQ in T2w. ME is generally safe and easy to use. However, self-administration in a hospital setting on-site before the MR exam might be stressful, leading to unsuccessful application and hence increasing the risk of incomplete bowel preparation. Administrating ME prior to the MR exam at home might be a way (i) for increasing the probability for a technically successful application and (ii) ensuring that ME can fully take effect before the scan. We acknowledge that there may be alternative approaches for artifact reduction [10]. However, ME is a bowel preparation technique for which available data (i) clearly indicates a positive effect on reduction of bowel peristalsis [18], (ii) strongly suggests improvement of DWI quality [5], (iii) where practically no side-effects are expected [30] and (iv) costs of the intervention are very low [5].

## Limitations

We recognize the following limitations. The patient count per cohort results in a moderate study power compared to some of the abovementioned studies [7, 11, 18]. When DR were introduced into clinical routine, HBB was already implemented, hence cohort/s of patients without HBB in combination with DR were not available. Moreover, when assessing the value of DR, a meaningful comparison was only possible between cohorts 3/3F and 4/4F, as otherwise > 1 parameter would have been altered between the cohorts and attribution of an observed effect to one of the bowel preparation techniques would be difficult. Finally, this study has investigated parameters of IQ between patients receiving different bowel preparation techniques. Therefore, studies investigating diagnostic accuracy will possibly put the potential benefits of bowel preparation techniques into perspective.

## Conclusion

In conclusion, Microenema seems to significantly improve image quality of DWI and the whole mpMRI image set of the prostate, while Hyoscine *N*-butylbromide and Dietary Restrictions did not show any benefit. Microenema’s safe and easy use, without risk of pharmacologic side-effects, make it a simple measure to improve image quality in prostate MRI.

## Abbreviations

mpMRI: Multiparametric Magnetic Resonance Imaging
CT: Computed tomography
DWI: Diffusion-weighted imaging
EPI: Echoplanar imaging
ADC: Apparent diffusion coefficient
DCE-MRI: Dynamic contrast-enhanced imaging MRI
HBB: Hyoscine *N*-Butylbromide
ME: Preparatory microenema
DR: Dietary restrictions
IQ: Image quality
PI-RADS: Prostate Imaging and Reporting Data System
ACR: American College of Radiology
ESUR: European Society of Urogenital Radiology
HIPAA: Health Insurance Portability and Accountability Act
Tp1, 2: Time Point 1, 2
ROI: Region of Interest
R1, 2, 3: Reader 1, 2, 3
Sc1, 2: MR Scanner 1, 2
